# Analysis of the Global Ocean Sampling (GOS) Project for Trends in Iron Uptake by Surface Ocean Microbes

**DOI:** 10.1371/journal.pone.0030931

**Published:** 2012-02-17

**Authors:** Eve Toulza, Alessandro Tagliabue, Stéphane Blain, Gwenael Piganeau

**Affiliations:** 1 UPMC Univ Paris 06, UMR 7232, Observatoire Océanologique, Banyuls-sur-Mer, France; 2 CNRS, UMR 7232, Observatoire Océanologique, Banyuls-sur-Mer, France; 3 IPSL/Laboratoire des Sciences du Climat et de l'Environnement, Gif-sur-Yvette, France; 4 CNRS, UMR 7621, Observatoire Océanologique, Banyuls-sur-mer, France; Universidad Miguel Hernandez, Spain

## Abstract

Microbial metagenomes are DNA samples of the most abundant, and therefore most successful organisms at the sampling time and location for a given cell size range. The study of microbial communities via their DNA content has revolutionized our understanding of microbial ecology and evolution. Iron availability is a critical resource that limits microbial communities' growth in many oceanic areas. Here, we built a database of 2319 sequences, corresponding to 140 gene families of iron metabolism with a large phylogenetic spread, to explore the microbial strategies of iron acquisition in the ocean's bacterial community. We estimate iron metabolism strategies from metagenome gene content and investigate whether their prevalence varies with dissolved iron concentrations obtained from a biogeochemical model. We show significant quantitative and qualitative variations in iron metabolism pathways, with a higher proportion of iron metabolism genes in low iron environments. We found a striking difference between coastal and open ocean sites regarding Fe^2+^ versus Fe^3+^ uptake gene prevalence. We also show that non-specific siderophore uptake increases in low iron open ocean environments, suggesting bacteria may acquire iron from natural siderophore-like organic complexes. Despite the lack of knowledge of iron uptake mechanisms in most marine microorganisms, our approach provides insights into how the iron metabolic pathways of microbial communities may vary with seawater iron concentrations.

## Introduction

Despite its high abundance in the Earth's crust, iron concentrations are very low in the ocean. This is due to the low solubility of iron in the oxic and slightly alkaline seawater of today's oceans, uptake by microorganisms and limited input from external sources. Consequently, the bioavailability of this element is very low in many oceanic regions [1]. Iron is essential for cell metabolism, especially for electron transport. Photosynthesis, respiration, and nitrogen fixation require high cellular concentrations of iron [2].

Microorganisms, particularly bacteria, have evolved several different iron uptake mechanisms [3], [4]. Inorganic iron can be acquired either in its reduced ferrous (Fe^2+^) [5], or oxidized ferric (Fe^3+^) state [6]. However, in well-oxygenated seawater, the free ion concentrations of both forms are low, and bacteria have developed alternative strategies to access the organically complexed pool. The synthesis and uptake of siderophores, strong chelators of ferric iron, is one example [7], but bacteria can also acquire iron from free heme (a prosthetic group of porphyrin containing an atom of iron) or heme-containing proteins using both direct uptake and hemophores [8]. Such strategies have been identified in diverse marine bacteria [9].

Many recent studies have highlighted the strategies used by diatoms and cyanobacteria to minimize their iron demand, such as reducing the expression of “expensive” iron genes in the photosystem I complex [10], [11] and nitrogenase [12], or else increasing flavodoxin production [13], [14]. Genomic analysis of *Prochlorococcus* clades isolated from iron-depleted oceanic regions showed that several genes encoding for iron-containing proteins were absent, thereby reducing the cellular iron quota [15].

The relation between genomic iron uptake strategies and oceanic environments has recently been investigated in cyanobacteria, the most abundant photosynthetic group of marine bacteria. Many *Synechococcus* genomes from strains isolated in the open ocean lack most known genes for iron stress, while genomes from strains isolated in coastal and upwelling areas contain many such genes, suggesting that maintaining multiple iron limitation compensation strategies is not a selective advantage in the open ocean [16]. Consistent with this, the light-harvesting gene *isiA* of *Synechococcus* has been proposed as a biomarker of HNLC regions [17]. However, heterotrophic bacteria also compete for iron at low concentrations, and can account for up to 50% of the total planktonic iron uptake [18]. Additionally, they may modify iron chemistry through the production of organic ligands and thereby regulating phytoplankton production [19]. Because metagenomes contain the gene content of the community of most abundant and therefore most successful microorganisms, they provide a complementary glimpse to organismal studies into the genetic basis of adaptation to the environment.

**Figure 1:**
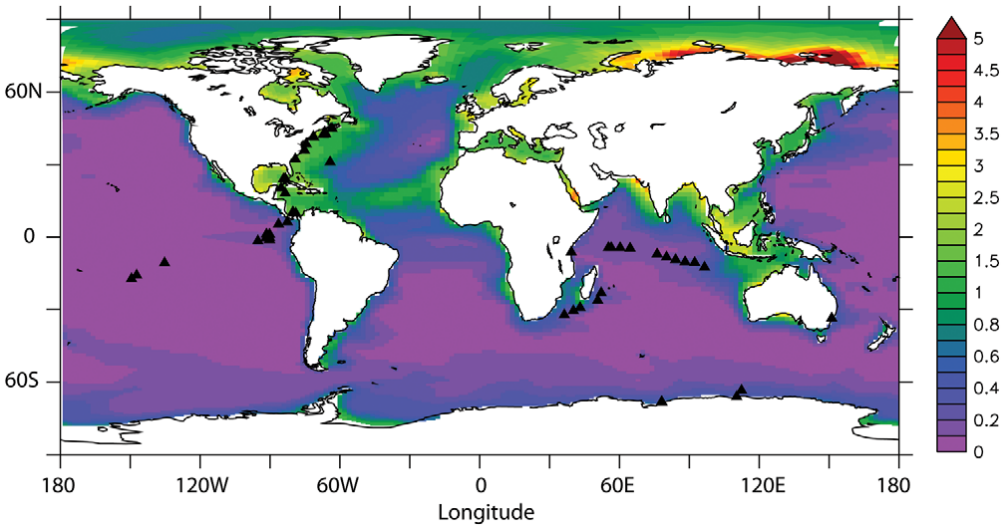
Map of annual average surface iron concentration (0–100 m) from the NEMO-PISCES model. Metagenomic sample sites are represented by black triangles. Color scale stands for dissolved iron concentration (nM).

We set up a database of 2319 sequences, corresponding to 140 gene families and 10 different iron-related metabolic pathways, to explore the mechanisms involved in iron acquisition in the ocean. The caveats of using Blast Best Hits and evalue cutoffs have been discussed previously [20]. Here, we defined a stringent criterion to discard false positives: we only considered Reciprocal Best Hits with coverage and identity threshold empirically estimated from our database. We investigate the link between genomic iron metabolism strategies of microbial communities and iron concentrations among 54 worldwide distributed marine metagenomes (Figure 1). Iron concentrations at each location were taken from a biogeochemical model that incorporates information about the sources and cycling of iron in the ocean and were compared to independently acquired observed iron concentrations. We thus established the relationships between microbial communities' iron-related gene prevalence, taxonomic affiliation and iron concentrations in different marine habitats.

## Methods

### Iron Metabolic Pathway database

We selected genes involved in iron metabolisms from the literature in cases where the protein product had been characterized or where the function of the protein could be inferred by sequence analysis. In this way, we identified 140 genes specifically involved in iron metabolism. We assigned these genes to 10 iron-related metabolic pathways, summarized in Table 1. A maximal phylogenetic coverage for each gene was achieved by retrieving all available bacterial sequences using the NCBI search tool with the gene names as a query. All 9917 annotations and protein sequences were manually inspected to discard irrelevant or incomplete protein sequences. To discard redundant sequences from the same genus, we randomly selected one full-size sequence per gene per available genus, resulting in 1753 remaining sequences. Additionally, we screened the annotations of the protein sequences from the Moore Microbial Genome database (marine bacteria isolates) for genes involved in iron metabolism (http://www.moore.org/microgenome/) and retrieved 566 genes. After manual inspection of these sequences, 191 putative ABC iron transporters could not be assigned to any one of the previous pathways, and therefore constituted the unspecified iron transport category (TR). We thus obtained a dataset of 2319 sequences belonging to 11 phyla (Actinobacteria, alphaproteobacteria, Bacteroidetes, betaproteobacteria, Cyanobacteria, deltaproteobacteria, Deinococcus-thermus, Firmicutes, gammaproteobacteria, Spirochetes, epsilonproteobacteria) (Table S1). The 140 gene families with at least two sequences were aligned and processed to estimate an identity and coverage threshold within each gene family. These thresholds estimations are needed because many proteins involved in different pathways could share sequence similarities, like ABC transporters of different iron-related pathways. We found that 65% amino-acid identity over a minimum length of 100 amino acids (or 80% of query length coverage) corresponded to 96% of correct orthologous gene assignment and 100% of correct pathway assignment (Table S2). Our criteria are stringent and we therefore probably underestimated the number of matches, but this enables a robust analysis of the different proportion of each iron-related pathway between sites.

**Table 1:** Iron-related metabolic pathway database.

Pathway	Abbreviation	Sequences	Genes in the database	Phylogenetic spread*	Genes with hits	Number of hits
Control (*recA*)	CN	120	1	11	1	4396
Flavodoxin switch [13]	FL	181	1	11	1	46
Fe^2+^ uptake [5]	F2	241	11	10	6	579
Fe^3+^ uptake [6]	F3	104	8	9	9	586
Heme uptake [8]	HE	196	24	10	9	23
Oxidative stress [56], [57]	OX	168	4	10	4	789
Regulation [58]	RG	269	9	11	6	602
Siderophore uptake	SU	562	23	10	6	620
Siderophore synthesis [7]	SS	164	56	7	2	5
Storage [48]	ST	123	3	9	3	139
Unspecified iron transport	TR	191	NA	6	35 sequences	3455
TOTAL		2319	140		47	11240

*number of most abundant phylogenetic groups represented (11 in total, see Methods).

### Metagenome Data and Screen

We analyzed metagenomic data from Global Ocean Sampling sites for organisms collected within a 0.1–0.8 µm size range. We downloaded data for metagenomes containing at least 50 million base pairs (Mbp) from the CAMERA database [21]. Sargasso Sea sample GS000A was discarded from the analysis as it is suspected of contamination [22]. We thus collected 54 metagenomes corresponding to 10.5 billion nucleotides from 4 Habitat types: 22 open ocean, 24 coastal, 4 coral reef, and 4 marine-derived lake (Antarctic) sites (Figure 1, Table S3). We screened these datasets with our gene database using TBLASTN [23]. Non-redundant hits were numbered based on reciprocal best hits [24] with our identity and coverage thresholds (Table S4). We used the gene encoding the recombinase A, RecA, as a single copy control to estimate taxonomic diversity. All metagenomic sequences where searched against a database of 120 RecA protein sequences with representatives in all prokaryotic taxonomic groups. A taxonomic group was considered to be present in a metagenome when a sequence had a reciprocal best hit with a *recA* gene belonging to this taxonomic group (Tatusov et al 1997). In a preliminary analysis, we first checked that the proportion of our control gene, *recA*, relative to hits against our iron-related gene database, did not vary with metagenome size (Spearman Rho = 0.03 p = 0.79). We checked the congruence of our *recA* based taxonomic affiliation with the 16S rRNA based taxonomic affiliation obtained on 29 metagenomes [25]. We found a very good correlation between phylum prevalence for both genes (Spearman correlation coefficient of 0.86, p<10–16). However, the variance in the number of hits within phylum was significantly lower for the single copy *recA* gene, which is expected as a consequence of 16S rDNA copy number variations. Total recruited reads per metabolic pathways were obtained by summing up results for all genes from the same pathway. We used hits to *recA* as a proxy for the number of microbial genomes and inferred taxonomic diversity from reciprocal blast best hits. *RecA* is assumed to be in single copy in most genomes and belongs to the class of housekeeping genes that does not frequently undergo horizontal gene transfer [26].

### Environmental Data

We extracted salinity, temperature, water depth and chlorophyll concentration for each sampling site from the CAMERA database [21]. We used the ocean general circulation and biogeochemistry model based NEMO-PISCES [27] to infer nitrate, phosphate and dissolved iron (dFe) concentrations. This resulted in 8 environmental variables per site (Table S3). Dissolved iron is an operational definition for the fraction of iron that passes through a 0.2 µm filter.

NEMO-PISCES simulates nanophytoplankton and diatoms, meso- and micro-zooplankton, small and large detritus, calcium carbonate, dissolved-inorganic-carbon, carbonate, dissolved-organic-carbon, oxygen, nitrate, phosphate, silicic acid, ammonium and dFe concentrations. The biotic iron demand varies between phytoplankton groups and as a function of dFe concentrations and light. dFe is removed via biotic uptake and particulate iron is remineralised back to dFe, with dFe scavenged as a function of the total particle load, with ligand complexation explicitly represented, assuming a uniform ligand concentration of 0.6 nM. For this study, we used a state-of-the-art version of NEMO-PISCES that includes aeolian, sedimentary, hydrothermal and fluvial dFe sources [27]. We verified the predictions of our model against the 2438 dFe observations (between 0 and 100 m) compiled in the database of Moore and Braucher [28]. We obtained a very good correlation (R^2^ = 0.56), with a bias of 0.09 nM between mean observed (0.32 nM) and modeled (0.23 nM) dFe (values compared at the same latitude, longitude, depth and month of sampling). Because our metagenomes all come from surface samples (<5 m) where dFe is expected to be more variable, we also checked the relationship between predicted and observed dFe for surface sites only (R^2^ = 0.54, Figure S1).

### Statistical analysis

All analyses were performed with R [29]. We first normalized the number of hits for each gene per site to the metagenome size in kbp. We checked that the proportion of iron-related metabolic pathways was not correlated to metagenome size (Spearman Rho = 0.11 p = 0.47).

Multivariate comparisons were performed with numerical ecology tools from the ade4 package [30]. We used non-parametric tests; Kruskal-Wallis test to assess significance levels of differences between habitats and Spearman's rank correlation coefficient to assess the significance of the relationship between metabolic pathways or gene prevalences and iron concentration. We used Fisher's combined probability X^2^ = −2*Σln(p) (which follows a Chisquare distribution with 2n degrees of freedom) to test the overall significance of several independent p-values bearing upon the same null hypothesis [31].

We followed the biogeography approaches [32], [33] to test for correlations between taxonomic distribution, iron metabolism pathways and environmental variables. The three datasets were represented with rows as sites, and columns were either proportion of metabolic pathways, proportion of different taxonomic groups based on *recA* assignation or environmental variables. We then constructed a matrix of iron content differences as distances between pairs of sites. We computed the three site-site correlation matrices by computing the Spearman's correlation coefficients between each pair of sites from each data frames. These correlation coefficient matrices were transformed in relative rank matrices: pairs of sites with higher correlation coefficients received lower ranks, whereas the pairs of site having less correlation received higher ranks [33]. The rank matrices thus contain the between site correlation ranks for iron metabolism pathways, taxonomy or environmental variables. We then estimated the between matrices correlation using a Mantel test to assess whether closely related sites on the basis of metabolic pathways were also closer in terms of taxonomic distribution or/and environmental variables or/and iron concentration.

### Iron Biomarker genes

The most abundant single genes of our database (*i.e.* 13 genes detected in at least 27/54 metagenomes) were tested individually for their distribution in habitats and their correlation with iron concentration. These genes were *bfr* (iron storage); *exbB, fur* (regulation); *fbpC, futA* (Fe^3+^ uptake); *feoB, yfeA, yfeB and yfeC* (Fe^2+^ uptake); *fecA* (siderophore uptake); *isiA, sodA, sodB* (protection against oxidative stress). We standardized the abundance of each gene against the number of single copy control *recA* gene per metagenome. We used Spearman's rank correlation in sites containing data for both iron-related pathway prevalence and dissolved iron concentrations.

## Results

### Iron-uptake gene prevalence depends on habitat types and is higher in low iron environments

**Figure 2:**
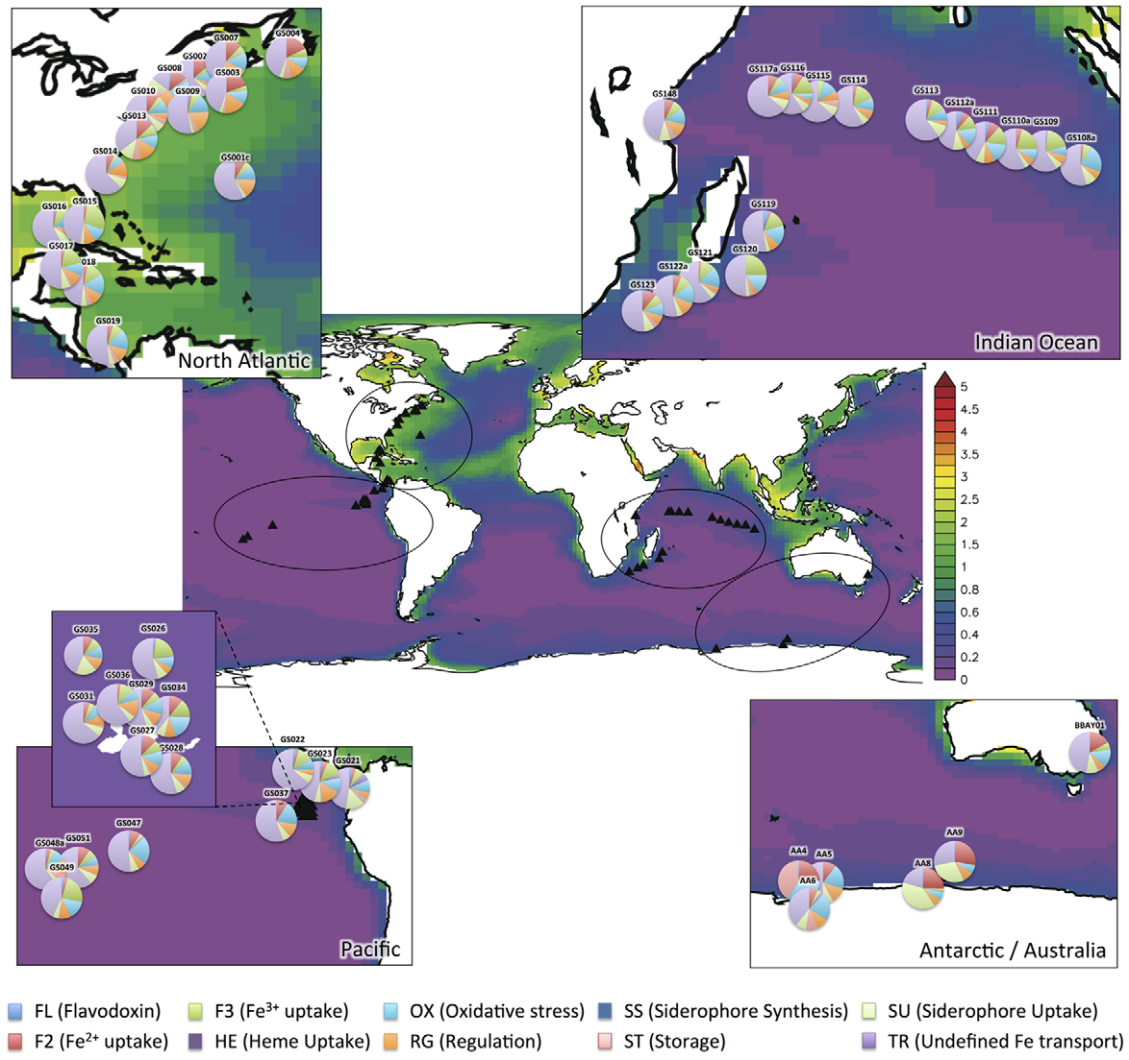
Proportion of iron-related metabolic pathways for each site overlaid on iron concentrations. Color scale stands for dissolved iron concentration (nM).

To screen marine metagenomes, we set up a database of 140 genes involved in 10 different pathways related to iron metabolism: flavodoxin switch, Fe2+ and Fe3+ uptake, heme uptake, response to oxidative stress, regulation, siderophore uptake, siderophore synthesis, storage, flavodoxin switch and unspecified iron transport (Table 1, Table S1). Microbial abundance and taxonomic diversity were inferred from the prevalence of the single copy *recA* gene. The 54 metagenomes larger than 50 Mbp have been obtained from 4 marine habitat types: coastal, open ocean, coral reef and marine-derived lakes (Figure S2, Table S3). First, we investigated whether the total proportion of iron related pathways varied between defined habitats. We found that the proportion of genes involved in iron metabolism pathways, relative to the control gene *recA*, is not equally distributed among habitat types (Kruskal-Wallis p = 0.002). Coastal sites contain a higher proportion of genes involved in iron metabolism, followed by coral reef, open ocean and marine-derived lake. This may reflect variations in the relative proportion of bacterial species, as compared to viral or picoeukaryotic communities, which have been shown to be present in 0.8 µm filtered metagenomes [34], [35]. Alternatively, this could reflect differences in iron-related gene copy numbers per genome between these habitats. Second, we investigated whether the proportion of iron-related pathways varied with predicted dissolved iron concentrations estimated from a biogeochemical model (Figure 2). Interestingly, the proportion of genes involved in iron metabolism pathways increases with decreasing iron concentration in open ocean and coastal habitats (Spearman Rho = −0.36, p = 0.014). This reflects a greater number of genes involved in iron uptake or metabolism in communities experiencing iron starvation. There is no significant correlation between the number of different iron-related pathways and iron concentrations. This suggests that the greater prevalence of iron-related genes in low iron environments is not the consequence of an increase in the number of different pathways, but the consequence of an increase in gene prevalence in several pathways.

Given this global trend, we investigated the extent to which each pathway prevalence varies between habitat. The prevalence of three iron related pathways varies between habitats (Table 2): the iron uptake pathways of Fe^2+^ and Fe^3+^, and the regulation pathway. Fe^2+^ uptake (F2) is under-represented in open ocean sites (Kruskal-Wallis p = 0.023), whereas Fe^3+^ uptake (F3) is over-represented in the open-ocean (Kruskal-Wallis p = 0.005). Regulation (RG) is over-represented in coral reef sites (Kruskal-Wallis p = 0.004). Since the prevalence of each pathway normalized by the metagenome size can be considered as independent, we can combine the probabilities across pathways to test whether there is a global difference of pathway prevalence between habitat types. Consistent with the analysis of total iron-related gene prevalence, the prevalence of iron-related metabolic pathways differs significantly between habitats (p<10^−5^). We also found that taxonomic prevalence is significantly different between habitat type (Table 2) and this prompted us to investigate the relationship between iron concentration and pathway prevalence in each habitat separately.

**Table 2:** Relationship between iron pathway prevalence, habitat and iron concentration across sites.

	Abb	Habitat effect Kruskal-Wallis p-value	Iron concentration effect Spearman Rho	
			Coastal sites	Open ocean sites
*Iron Pathway Prevalence*				
Flavodoxin Switch	FL	0.132	0.04	**−0.45** *
Fe^2+^ uptake	F2	**0.023**	−0.33	−0.26
Fe^3+^ uptake	F3	**0.005**	−0.23	0.06
Heme uptake	HE	0.474	0.04	**−0.57** **
Oxidative stress	OX	0.262	−0.32	−0.21
Regulation	RG	**0.004**	−0.29	0.02
Siderophore synthesis	SS	0.324	0.35	−0.26
Storage	ST	**0.028**	0.31	**0.56** **
Siderophore uptake	SU	**0.044**	−0.14	**−0.53** *
Unspecified iron transport	TR	**0.003**	**−0.49** *	−0.31
Fisher combined p-value		***	*	***
*Taxonomic prevalence*				
Actinobacteria	AB	0.951	0.34	−0.38
Bacteroidetes	BA	0.521	−0.19	0.20
Cyanobacteria	CY	**9.10^−5^**	0.11	0.13
Deinococcus-Thermus	DT	0.862	−0.12	0.22
Firmicutes	FI	0.12	0.24	0.26
Alpha-proteobacteria	aP	**0.010**	0.20	−0.06
Beta-Proteobacteria	bP	0.783	0.34	0.04
Delta-Proteobacteria	dP	0.630	−0.17	**0.51** *
Epsilon-Proteobacteria	eP	**0.025**	−0.30	nd
Gamma-Proteobacteria	gP	**0.015**	0.05	−0.15
Spirochetes	SP	0.428	0.13	−0.003
Fisher combined p-value		***	**ns**	**ns**

*: p<0.05,

**: p<0.01,

***: p<0.001,

**ns: not significant.**

### Iron-related gene prevalence reveal biological adaptations to iron concentrations

To explore whether microbial communities have different strategies of iron metabolism as a consequence of iron availability, we investigated the statistical significance of the variation between the relative proportion of each of the 10 iron-related pathways and iron concentration for 23 coastal and 22 open ocean sites, where iron concentrations can be inferred from the biogeochemical model. While there is a globally significant correlation between pathway prevalence and iron concentrations (combined probabilities across pathways 0.038 and 0.006 for coastal and open ocean environments, respectively), most individually significant trends appear in the open ocean habitat (Table 2). Storage prevalence increases significantly with predicted iron concentration (Spearman Rho = 0.56 p = 0.007) (Table 2) whereas the siderophore uptake pathway decreases with increasing iron concentration in open ocean sites (Spearman Rho = −0.53 p = 0.011), suggesting that this is an iron starvation strategy in this habitat. Consistent with this idea, experimental evidence suggests that siderophore synthesis does not occur in iron-rich media for many species [36]. Although more rarely detected, heme uptake and flavodoxin switch show negative correlations with iron concentration in open ocean sites (Spearman Rho = −0.57, p = 0.006 and Spearman Rho = −0.45, p = 0.034, respectively).

**Figure 3:**
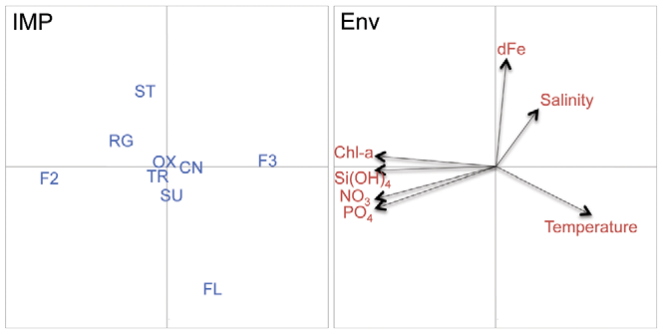
Scatter diagram of Canonical Correspondence Analysis. The iron-related metabolic pathways and environmental variables are projected as a result of CCA on 37 metagenomes. Left panel: position of iron-related metabolic pathways (IMP) on the canonical axes. Right panel: contribution of the environmental variables to the canonical space. (CN: control *recA*; F2: Fe^2+^ uptake; F3: Fe^3+^ uptake; FL: flavodoxin switch; OX: oxidative stress; RG: regulation; ST: storage; SU: siderophore uptake; TR: unspecified iron transport).

These correlations between different iron metabolism pathways and environmental variables are summarized in the canonical correlation analysis scattergram based on iron-related pathways proportion between sites (Figure 3). This analysis highlights two pairs of anti-correlated iron uptake strategies: Fe^2+^ uptake (F2) versus Fe^3+^ uptake (F3) (Spearman Rho = −0.47, p-value = 0.002) and storage (ST) versus flavodoxin switch (FL) (Rho −0.38, p = 0.015) and provides a further illustration of the positive correlation of storage (ST) with iron concentration. Nutrient concentrations (phosphate, silicate and nitrate) are correlated with each other and with Fe^2+^ uptake, which is typical of coastal sites, and negatively correlated with Fe^3+^ uptake, which is typical of open ocean sites. The concentration of these nutrients is not significantly correlated to iron concentrations.

### Taxonomic diversity and iron-related metabolic pathway prevalence covary between but not within habitats

In order to assess the relative importance of taxonomy in the observed correlation between dFe and iron metabolism pathway prevalence, we investigated the relationship between phylum prevalence and dissolved iron contration (dFe) (Table 2). Only one minor class (deltaproteobacteria) shows a significant relationship with dFe in open ocean. In a previous analysis of 16S rDNA prevalence [25], deltaproteobacteria were also poorly represented and mainly affiliated to the SAR324 clade. Overall, there is no significant relationship between phylum prevalence and dFe (Fisher exact test for coastal and open ocean sites). There is, however, a striking difference in taxonomic diversity between habitats.

To further test global correlations between iron-related metabolic pathway (IMP) prevalence, environmental variables and taxonomy, we followed classical biogeography analyses [32], [37] (see Methods). Essentially, we tested whether between site correlations, estimated from three different matrices (environmental variables, iron metabolism pathways prevalence and taxonomic diversity) showed a similar pattern. This enables to assess the strength of the possible covariations between environmental factors, taxonomic diversity, and functional diversity. The null hypothesis we test here is that there is no covariation, *i.e.* sites are correlated independently by any of the pair of matrices compared.

We performed these analyses on the 37 sites for which all 8 environmental variables were available: 20 open ocean, 15 coastal and 2 coral reef sites. We found a correlation between the prevalence of iron-related pathways and taxonomy between sites (Mantel test Rho = 0.19, p = 0.001). Environmental correlations were associated to iron metabolism pathways (Mantel test, Rho = 0.11 p = 0.008) and to taxonomy (Mantel test, Rho = 0.24, p = 0.001). Thus, we detected a significant correlation between pathway prevalence (functional diversity), taxonomy and environmental variables.

We then examined whether it was possible to detect a specific effect of iron concentration on iron metabolism pathway prevalence and taxonomy by taking iron content differences as distances between pairs of sites (Table 3). The taxonomy matrices and iron metabolism matrices were correlated (Mantel test, Rho = 0.38, p = 0.001) for the 45 sites for which dissolved iron concentration could be inferred (22 open ocean and 23 coastal sites). Iron concentration differences were weakly associated to iron-related pathway prevalences (Mantel test, Rho = 0.06, p = 0.043), whereas there was no correlation between iron concentration differences and taxonomy (Mantel test, Rho = 0.05, p = 0.092). Because taxonomy and pathway prevalence both vary with habitat type, the habitat type may explain part of the above correlations between sites. We therefore analyzed the dependency on iron concentrations in open ocean and coastal sites independently. In both habitats, taxonomy at the phylum level and iron-related metabolic pathways remain significantly correlated (Mantel test, open ocean Rho = 0.27, coastal Rho = 0.34, p = 0.001). Within open ocean sites, the correlations between iron concentration and iron metabolism pathways or taxonomy are no longer significant, probably because variation in iron concentration was too low within open ocean sites. However, in coastal sites, iron concentration and iron metabolism pathways remain significantly correlated (Mantel test, Rho = 0.14, p = 0.02). However, iron concentrations are no longer correlated to taxonomic diversity.

**Table 3:** Relationship between iron pathway prevalence, taxonomy and iron differences between pairs of sites.

	All sites	Coastal sites	Open Ocean sites
Number of sites	45	22	23
IMP×dFe	0.06*	0.14*	ns
IMP×Taxo	0.38**	0.34**	0.27**
Taxo×dFe	ns	ns	ns

*: p<0.05,

**: p<0.01,

***: p<0.001,

**ns: not significant.**

### Iron Biomarker Genes

To identify possible iron biomarker genes, we tested the relationship between the prevalence of the 13 most represented single genes of our database (*i.e.* detected in at least half of the 54 metagenomes screened) and iron concentration using Spearman's rank coefficient for sites where the gene was detected. *Bfr* (bacterioferritin) showed a strong positive correlation with predicted dissolved iron (Rho = 0.50, p = 0.007). Conversely, *fecA* (encoding the ferric dicitrate outer membrane transporter) was negatively correlated with iron concentration (Rho = −0.31, p = 0.03). In open ocean sites, this correlation was much stronger (Rho = −0.61, p = 0.002). We found no significant correlation between the prevalence of the cyanobacterial photosystem *isiA* gene and predicted iron concentrations. This gene is encoding the iron-stress chlorophyll-binding protein and had previously been suggested as an iron biomarker gene [17]. However, the prevalence of *isiA* is significantly higher in open ocean sites as opposed to coastal sites (Kruskal-Wallis p = 0.004).

## Discussion

We show significant quantitative and qualitative variations in iron-related strategies with predicted iron concentrations, and a global trend of increasing proportions of iron uptake genes in low iron environments. The sign of observed correlations are consistent with results obtained from experimental studies: the iron storage pathway prevalence increases with simulated dissolved iron concentrations (Rho = 0.56), whereas siderophore uptake prevalence appears to be a low iron strategy (Rho = −0.53). A striking qualitative difference in iron uptake strategies between coastal and open ocean habitats is the negative correlation between Fe^2+^ uptake versus Fe^3+^ uptake. The bioavailability of iron in the ocean is linked to its chemical speciation, which is not well known. Most of dissolved iron is complexed by organic ligands (like siderophores); however, inorganic species also exist, though at much lower concentrations. The fraction of “free” iron, despite its extremely low steady state concentration, is thus an important resource for bacteria. This is consistent both with field data on iron speciation, which shows that unchelated iron can be an important source of iron to the phytoplankton in the sea [38], and with gene content analysis of marine cyanobacteria, suggesting that some strains are specialized in one ferric state uptake [39]. Fe^2+^ uptake prevalence is indeed significantly higher in coastal sites, whereas Fe^3+^ uptake is higher in the open ocean. Consistently, there is a significant relationship between the proportion of cyanobacteria and the habitat (Table 2). The prevalence of Fe^2+^ at a given region might result from elevated photoreduction of organically complexed Fe^3+^ (i.e. a greater source) which can be elevated in coastal sites [40] or reduced Fe^2+^ oxidation (i.e. reduced sink) due, for example, to low oxygen microenvironment arising from large particles or aggregates that are abundant in coastal waters [41]. We speculate that the striking differences between genomic prevalence of Fe^2+^ and Fe^3+^ uptake genes in open ocean *versus* coastal environments could reflect an as yet unreported bioavailability difference in these two oxidative states of iron. A better knowledge of the bioavailability of these inorganic forms will require precise determination of the supply rate in different environmental conditions.

From the 10 pathways identified from the literature, two pathways have very few sequence representatives in the metagenomes. The first rare pathway is heme uptake (9 genes of this pathway have at least one hit in 11 metagenomes), consistent with recent evidence that many free living marine bacteria lack orthologous genes of this pathway [9]. The second rare pathway is siderophore synthesis (detected in 3 of 54 sites), whereas siderophore uptake was present in all metagenomes screened. There are two kinds of hypotheses to explain this apparent paradox. First, genes involved in siderophore synthesis may be more species-specific than those involved in siderophore uptake, such that our similarity based approach cannot efficiently detect genes involved in siderophore synthesis. Siderophore synthesis is performed by nonribosomal peptide synthetases (NRPS) or NRPS-independent pathways, the latter pathway being much less well characterized [42]. Moreover, these biosynthesis pathways are very diverse and specific for each type of siderophore. In contrast, all siderophore receptor systems identified so far are composed of a specific membrane siderophore receptor and periplasmic binding proteins (Gram negative), and ABC-type transport proteins, showing many structural similarities [7]. Consistent with this, siderophore uptake is represented by fewer genes in our database (23), with an average of 15.6 sequences per gene and an average of 4 taxonomic groups per gene, whereas siderophore synthesis is represented by more genes (53) with an average of 3.8 sequences per gene and an average of 2.1 taxonomic groups per gene. Both differences in diversity are significant in terms of the average number of sequences (Wilcoxon test, p = 0.0004), and the number of taxonomic groups per gene (Wilcoxon test, p = 0.0003). The higher diversification of the siderophore synthesis gene family might thus explain the few number of hits observed. A second explanation could be that specific siderophore synthesis is not an evolutionary stable strategy [43] in the ocean, as it is too expensive and wasteful for marine microorganisms, and therefore those microorganisms producing siderophores are counter-selected. In contrast, the siderophore uptake strategy is advantageous, because “natural” siderophores, such as citrate, which is a metabolic byproduct, are present in the environment and can be taken up by siderophore uptake genes like the receptor *fecA*. Marine bacteria may thus take advantage of the presence of siderophore-iron complexes, which are not necessarily excreted by bacteria to the marine environment.

This “natural” siderophore uptake gene, *fecA*, is one of the two candidate genes for iron bioavailability we have identified, based on a significant correlation between their prevalence and predicted iron concentration. *FecA* is the outer membrane receptor component of ABC-transporter of dicitrate-type siderophores [44]. One of the main structural features of marine siderophores identified so far is that they contain predominantly α-hydroxy-carboxylic acids (like citric-acid) [45]. These are photoreactive siderophores such as petrobactin, ochrobactins, synechobactins, alterobactin, or dicitrate itself. In addition, in dilute environments like seawater where the synthesis of specific siderophores may be prohibitively wasteful for isolated cells, iron may be complexed to natural organic ligands [46]. Citrate is ubiquitous in nature and can complex Fe^3+^ in the form of ferric-dicitrate [47]. Our results suggest that uptake of ferric-dicitrate as a source of iron may be particularly important in open ocean waters. Since *fecA* is absent from cyanobacterial genomes, this gene is a good candidate for iron bioavailability for the heterotrophic bacterial community. This will have to be investigated experimentally in the ocean, e.g. with quantitative PCR.

The second candidate gene for iron bioavailability, bacterioferritin (*bfr*) is involved in iron storage inside the cell [48] and we found that its prevalence increases with predicted iron concentration. This positive relationship is consistent with a recent proteomic analysis in *Acetinobacter* that shows that it is upregulated in iron-rich culture conditions compared to iron-chelated media [49]. Moreover, expansion in the number of *bfr* copies in *Synechococcus* genome is associated with coastal environments [39].

All previous pan-oceanic metagenomic studies have evidenced a strong habitat effect [15], [17], [33], [37]. This is consistent with the large body of work from both geochemical and microbial biodiversity surveys. Coastal sites are nutrient-rich because of the proximity of land-based sources and account for approximately 30% of all marine biological productivity [50]. The open-ocean is typically more stable and generally poorer in nutrients with lower biomass levels, but with a remarkably high diversity.

Horizontal Gene Transfer (HGT), which is the exchange of genes between distantly related bacteria, is a major mechanism of genome evolution in prokaryotes [51], [52]. Not surprisingly, HGT has been found to be prevalent in marine bacterial genome evolution [53] and especially so for transporter genes [32], [54]. Therefore, one might expect that microbial communities' taxonomic and functional diversity are not strongly correlated. Consistent with this, previous studies have shown that there is a much stronger correlation between functional diversity, measured as membrane protein diversity, and environmental variables compared with that between taxonomic diversity, measured from 16S rDNA, and environmental variables for 29 metagenomes [33], [55]. Our results suggest that both iron-related metabolic pathway prevalence and taxonomy at the phylum level are correlated with dissolved iron concentrations, suggesting an important phylogenetic inertia between functional and taxonomic diversity (at the phylum level) on iron metabolism genes.

In conclusion, marine metagenomes enable us to investigate how growth limiting abiotic factors may shape the most abundant, and therefore most successful, genes in a community assemblage. Here, we show that different iron metabolism strategies, inferred from gene prevalence, vary with iron concentrations across marine environments, and that both habitat type and taxonomy are important factors to take into account at a global scale. Our analysis indicates that iron storage (especially bacterioferritin) and flavodoxin switch are the most prevalent iron response strategies, whereas siderophore uptake (especially the ferric-dicitrate receptor gene *fecA*) increases with iron depletion in the open ocean. The difference between Fe^2+^ and Fe^3+^ uptake between coastal and open ocean environments and the unexpected prevalence of dicitrate receptors shed new light on the bioavailability of iron for bacteria in the marine environment.

## Supporting Information

Figure S1 **Relationship between predicted and observed dFe for surface sites.**
(TIF)

Figure S2 **Proportion of iron-related metabolic pathways between habitats.** Frequencies are relative to the number of control gene hits (*recA*) for each site.
(TIF)

Table S1 **Database of iron-related genes.**
(XLS)

Table S2 **Identity between gene families in the database.**
(XLS)

Table S3 **Metagenomes and associated environmental variables.**
(XLS)

Table S4 **Number of RecA and iron metabolism genes for each metagenome.**
(XLS)
