# Supplementary figures and images for: Analysis of the Global Ocean Sampling (GOS) Project for Trends in Iron Uptake by Surface Ocean Microbes
Source: PLoS One. 2012 Feb 17;7(2):e30931. doi: 10.1371/journal.pone.0030931 (PMC3281889; doi:10.1371/journal.pone.0030931)

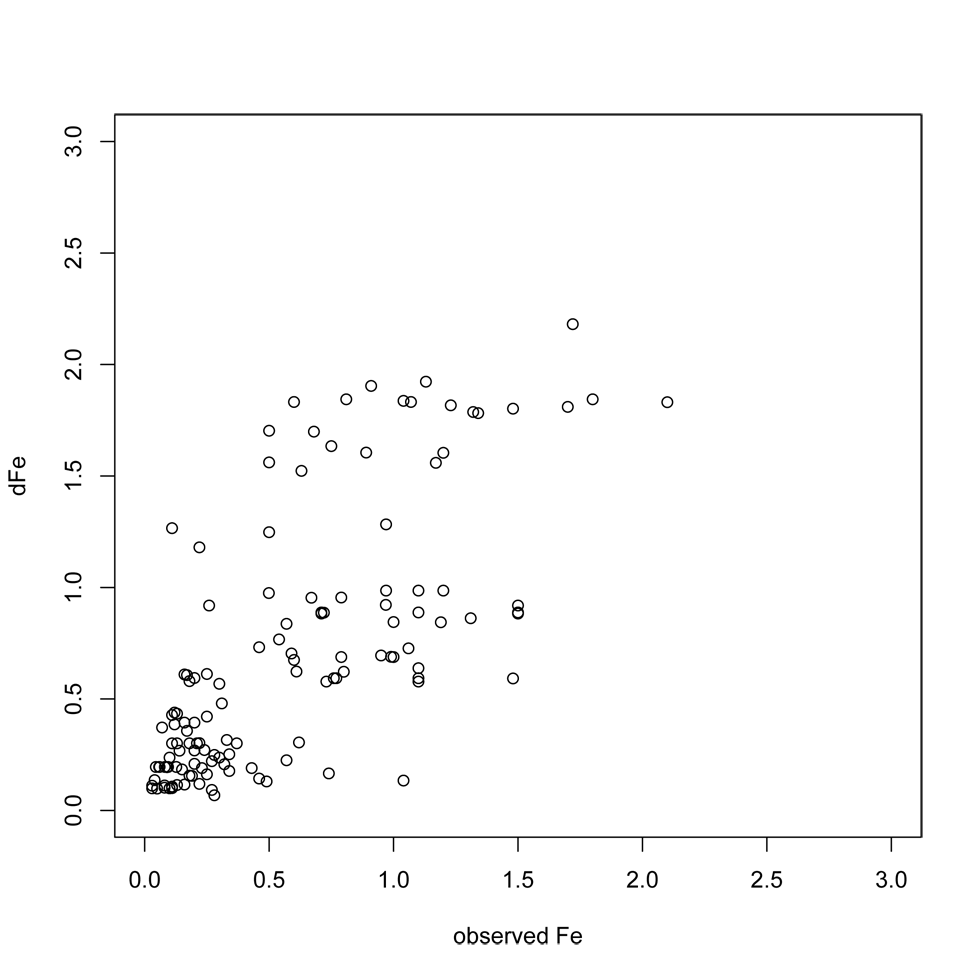

Supplement: Figure S1 — Relationship between predicted and observed dFe for surface sites. (TIF) [file pone.0030931.s001.tif]

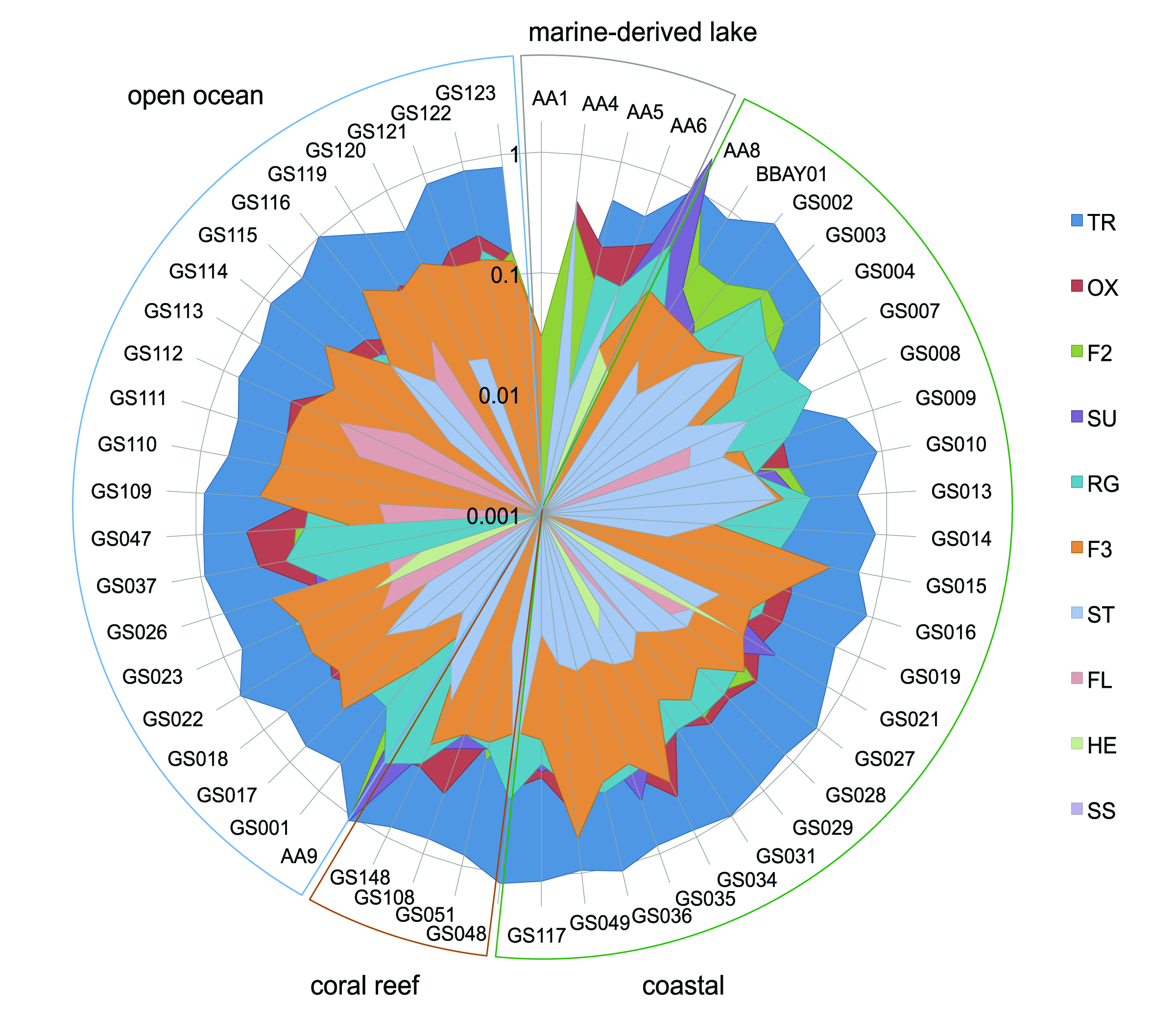

Supplement: Figure S2 — Proportion of iron-related metabolic pathways between habitats. Frequencies are relative to the number of control gene hits (recA) for each site. (TIF) [file pone.0030931.s002.tif]
